# Fatal Tension Pneumoperitoneum Due to Non-Accidental Trauma

**DOI:** 10.5811/westjem.2015.7.28022

**Published:** 2015-10-20

**Authors:** Stephen L. Thornton, Jeremy Hunter, Mark Scott

**Affiliations:** Department of Emergency Medicine, University of Kansas Hospital, Kansas City, Kansas

A previously healthy two-year-old boy presented to the emergency department with vomiting. He was cyanotic with mottling of both lower extremities. He was in respiratory distress with retractions and diminished breath sounds. His abdomen was distended and rigid. He had a pulse of 170 beats per minute, blood pressure of 144/69mmHg and respiratory rate of 42 breaths per minute. He was endotracheally intubated. Chest and abdominal radiographs demonstrated a tension pneumoperitoneum (Figure 1).

Abdominal decompression was performed with a 16-gauge needle in the left lower quadrant. Bilateral tube thoracotomies were also performed. Post-decompression radiograph demonstrated continued free air but normal lie of organs and viscera (Figure 2). The patient then went into cardiopulmonary arrest. Chest compressions, epinephrine, bicarbonate, atropine and calcium gluconate were administered, but he did not regain spontaneous circulation. Subsequent autopsy and investigation determined the patient had been a victim of non-accidental trauma resulting in gastric rupture.

In pediatric patients tension pneumoperitoneum is a rare complication described after reduction of intussusceptions, mouth-to-mouth breathing, iatrogenic bowel perforations, and positive pressure ventilation.^1^–^4^ It has not been described as a complication of non-accidental trauma. The increase in intra-abdominal pressure causes multiple physiologic derangements including decreased cardiac return via compression of the inferior vena cave and respiratory failure due to splinting of the diaphragms.^3^

Initial symptoms include abdominal pain and distension followed by hypoxia and shock.^1^–^4^ Diagnosis is clinical, but radiographs will demonstrate free air and medial displacement of the solid organs and viscera.

If not recognized and promptly treated tension pneumoperitoneum can rapidly lead to cardiopulmonary arrest. Treatment is emergent needle decompression followed by definitive laparotomy repair.^4^ Emergency medicine clinicians should be familiar with tension pneumoperitoneum as a cause of respiratory distress and cardiovascular collapse in the pediatric patient, as early recognition and treatment is critical in improving survival.

## Figures and Tables

**Figure 1:**
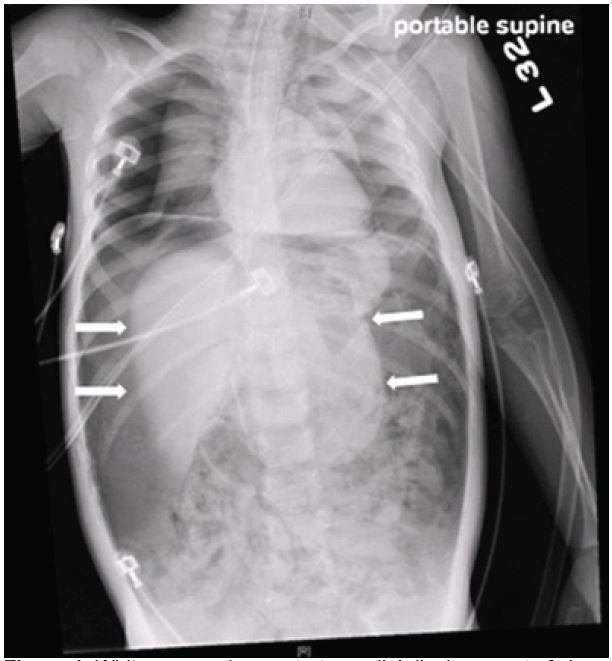
White arrows demonstrate medial displacement of viscera. Free air is present.

**Figure 2:**
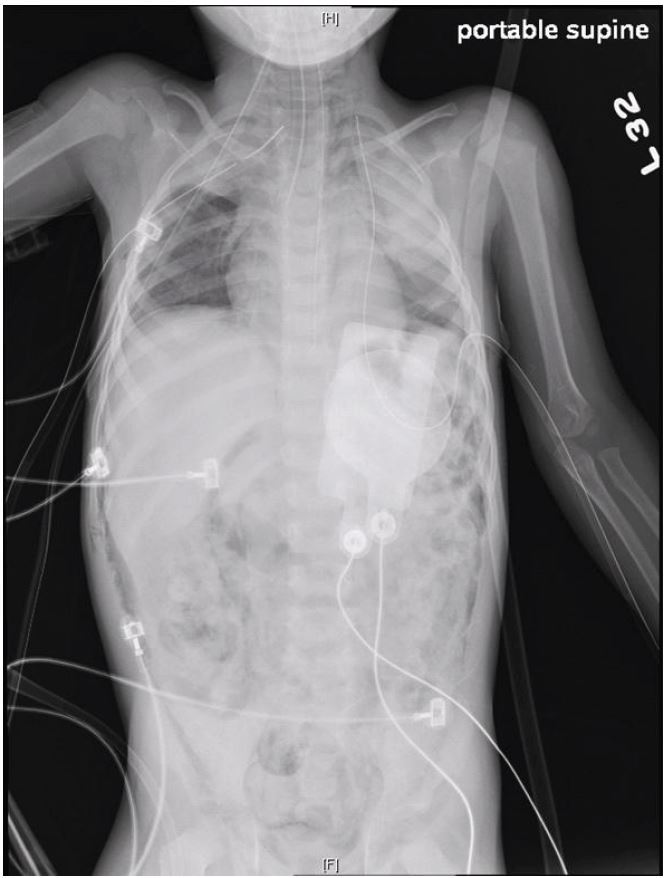
After decompression viscera demonstrate normal lie. Free air is still present.
